# Reversal Strategies for Intracranial Hemorrhage Related to Direct Oral Anticoagulant Medications

**DOI:** 10.1155/2018/4907164

**Published:** 2018-07-04

**Authors:** Alok Dabi, Aristides P. Koutrouvelis

**Affiliations:** ^1^Neurosciences Critical Care Program, University of Texas Medical Branch (UTMB), Galveston, TX 77555, USA; ^2^Department of Anesthesiology, Anesthesiology Critical Care Medicine, Surgical and Trauma Intensive Care Unit, Galveston, TX 77555, USA

## Abstract

Direct oral anticoagulants (DOACs) are a new class of anticoagulants that directly inhibit either thrombin or factor Xa in the coagulation cascade. They are being increasingly used instead of warfarin or other vitamin K antagonists (VKAs). Adverse side effects of DOACs may result in hemorrhagic complications, including life-threatening intracranial hemorrhage (ICH), though to a much lesser degree than VKAs. Currently there are relatively limited indications for DOACS but their usage is certain to expand with the availability of their respective specific reversal agents. Currently, only idarucizumab (antidote for dabigatran) has been United States Food and Drug Administration- (FDA-) approved, but others (andexanet-*α* and ciraparantag) may be approved in near future, and the development and availability of such reversal agents have the potential to dramatically change the current anticoagulant use by providing reversal of multiple oral anticoagulants. Until all the DOACs have FDA-approved reversal agents, the treatment of the dreaded side effects of bleeding is challenging. This article is an attempt to provide an overview of the management of hemorrhage, especially ICH, related to DOAC use.

## 1. Introduction

Intracerebral hemorrhage (ICH) is a nontraumatic brain parenchymal hemorrhage, a stroke subtype, that may extend into the ventricular system or into the subarachnoid space [1]. Other types of intracranial hemorrhages are epidural, subdural, and subarachnoid hemorrhage, most commonly caused by trauma.

The annual incidence of ICH is 16 to 33 cases per 100,000 general population [2]. In 2010, there were an estimated 5.3 million cases globally, with more than 3.0 million deaths [3]. Despite the relatively low incidence, ICH is responsible for the majority of the stroke mortality, with case-fatality rate ranging from 35% at 7 days to 59% at one year, with half of the fatalities occurring in the first 48 hours of onset [4]. ICH survivors are usually left with severe disability, and only about 40% of them achieve partial functional independence about a year later [1, 5].

Though hypertension and cerebral amyloid angiopathy contribute to the vast majority of ICH incidence, in recent times, the anticoagulant therapy has been recognized as a small but significant avoidable cause of ICH. Amongst the anticoagulant medications, vitamin K antagonists (VKAs), such as warfarin, as well as other VKAs, have been traditionally considered the principle offenders. Historically in 1938, 3,3′-methylenebis-(4-hydroxycoumarin), a congener of warfarin, was first discovered in spoiled sweet clover ingested by Wisconsin cows. Warfarin was then used as a rodenticide, with later use in human cases, including President Eisenhower as an anticoagulant in the 1950s [6]. However, recently warfarin has been superseded by the newer medications, collectively called direct oral anticoagulants (DOACs), approved for nonvalvular atrial fibrillation because of its efficacy and improved side effect profile regarding intracerebral bleeding.

**Figure 1:**
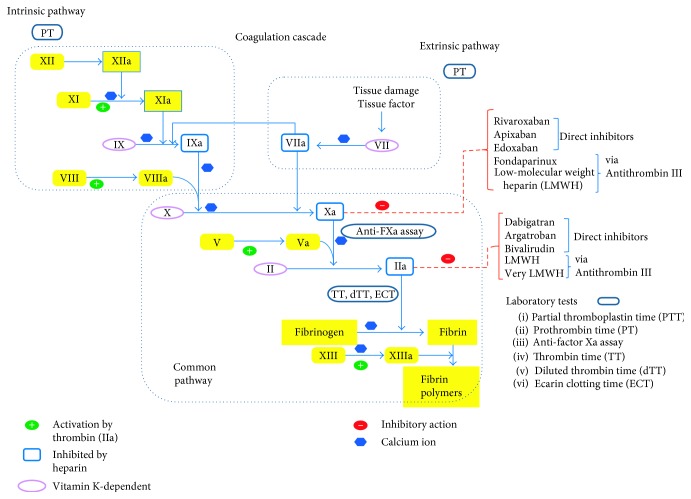
Coagulation cascade.

DOACs fall into 2 categories—factor IIa (thrombin) inhibitor (dabigatran) and factor Xa inhibitors (rivaroxaban, apixaban, edoxaban, and betrixaban). Direct thrombin inhibitor, hirudin, was first isolated from leech saliva, whereas factor Xa inhibitor, TIX-5, was first discovered from tick saliva [7]. The first medication of the DOAC group to be approved by the United States Food and Drug Administration (FDA) was dabigatran etexilate (Pradaxa) (Boehringer Ingelheim Pharmaceuticals, Inc.) in October 2010, and this is a direct thrombin inhibitor. This was quickly followed by rivaroxaban (Xarelto) FDA approval in July 2011 (Janssen Pharmaceuticals, Inc.) and then by apixaban (Eliquis) in December 2012 (Bristol-Myers Squibb Company and Pfizer Inc.). Relatively recently, edoxaban (Savaysa) by Daiichi Sankyo, Japan, has been FDA-approved in January 2015. Another DOAC called betrixaban (Bevyxxa, Portola Pharmaceuticals, Inc. California, USA) got FDA approval in June 2017 [8] (Figure 1). The DOACs which are either Xa inhibitors or direct thrombin inhibitors are oral agents. Unlike dabigatran, a direct thrombin inhibitor, at present, there is no reversal agent for the Xa inhibitors.

This new category of drugs, that is, DOACs, provides many advantages over VKAs. With warfarin, the disadvantages are as follows: wide array of pharmacokinetic variability, the multiple drug-drug interactions, and the need for restrictions on diet and alcohol consumption, and consequently the need for frequent blood monitoring of INR, a normalized ratio of prothrombin time, which measures the narrow therapeutic window of warfarin's efficacy. It has been found that warfarin stays within the therapeutic range rarely above 65% of the duration of the therapy [9].

DOACs have been found to have about 50% lower chances of ICH than warfarin, with a lower incidence of hemorrhage in all other major bleeding sites with the only exception being the gastrointestinal (GI) tract. Their convenient fixed dosing, rapid onset of action, short half-lives, more predictable pharmacokinetics, and relative lack of drug and food interactions make them an attractive alternative to warfarin and other VKAs. Most current guidelines in USA, Canada, and Europe now prefer DOACs over the VKAs for stroke prevention in nonvalvular atrial fibrillation (AF) and venous thromboembolic (VTE) treatment and prophylaxis in patients without active cancer. Current DOACs (except edoxaban and betrixaban) are also approved for thromboprophylaxis after elective hip or knee surgery [10]. With all these advantages, not surprisingly, the use of DOACs has increased, particularly in the United States and Canada [11]. With increasing number of DOAC prescriptions, certain concerns have also been raised about their use, namely, higher drug costs, lack of a specific reversal agent, lack of available laboratory monitoring of the level of anticoagulation provided, and their use in patients with renal dysfunction. Therefore, physicians are now encountering increasing number of patients with hemorrhages, including the life-threatening ICH, associated with the use of DOACs (Table 1) [12].

### 1.1. Intracranial Hemorrhage (ICH) with the Use of DOACs

Incidence of major bleeding with the use of DOACs is about 3-4% of the patients per year [16]. ICHs comprise about 13% of all major bleeds in all DOAC-treated patients, with annual rates ranging from 8 to 16%, whereas gastrointestinal (GI) hemorrhage constitutes above 50% of all major bleeding events [17]. The unusually high prevalence of GI hemorrhage with DOACs is thought to be due to relative lack of GI absorption leading to increased local drug level, with subsequent mucosal hemorrhage.

More than 900 ICH cases are associated with factor Xa inhibitors each month in the United States. Importantly, even this rate of hemorrhagic complications associated with the use of DOACs is either equivalent or significantly lower than that of warfarin. Major bleeding events, however, do increase the risk of mortality. Of all the types of bleeding complications from DOACs, the ICH leads to most cases of mortality accounting for up to 45% of all the bleeding-related deaths. Amongst all DOAC-related major bleeding events, ICHs accounted for about 11% and was associated with a 4-fold increased risk of mortality, as compared to other extracranial major bleeds [18].

The hemorrhagic complications due to anticoagulant medications lead to significant additional health-care costs. These patients need to be admitted to hospital, mostly to the intensive care units, with a need to be attended by additional physician specialists, with a median number of up to 4 specialist consult encounters per admission. Among all hospital admissions related to major hemorrhagic events, independent of the site of bleed, the average patient length of stay was about 10 days, with the mean total health-care cost per patient of about $60,000. Total all-cause health-care cost during the first 12 months of follow-up for patients with atrial fibrillation, with major bleeding, was almost double the amount compared to patients without major bleeding (about $64,000 versus about $38,000) [19]. Unfortunately, patients who experience a major hemorrhagic complication on the DOACs or warfarin use are also at a higher risk of developing subsequent thromboembolic events. The rate of venous thromboembolic events may range from 7 to 12% within 30 days of a DOAC-associated major hemorrhagic complication [20]. This study did not look into the occurrence of thromboembolic complication whether the patient was on or off anticoagulation in the postbleed period.

### 1.2. Optimal DOAC Selection

Use of DOACs is contraindicated for patients with mechanical heart valves, and it is not recommended in severe renal insufficiency, patients with known cancer, and if there is concern for cost and drug compliance. DOACs should be avoided in patients with a body mass index above 40 kg/m [2] or those with body weight of over 120 kg [21]. Dabigatran, in particular, should additionally be avoided in moderate renal insufficiency, but is preferred in patients with a high stroke risk (when used at 150 mg twice a day dose) without renal dysfunction. Rivaroxaban or edoxaban are preferred when once daily dosing is needed, and edoxaban is preferred if the risk of pulmonary embolism is high (Table 1) [22].

### 1.3. Laboratory Monitoring of Direct Oral Anticoagulants

Routine use of DOACs does not require routine monitoring of the anticoagulant effect, but in certain clinical situations, monitoring is critical, for example, urgent or emergent surgery, assessing medication compliance, or patients at the extremes of the body weight. Commonly implemented coagulation-related laboratory tests, such as the prothrombin time (PT), international normalized ratio (INR), and activated partial thromboplastin time (aPTT) may not accurately reflect the clinical efficacy while being prescribed DOACs. Thrombin time (TT) has been used for detecting the presence of dabigatran, but a modified version of TT; the dilute thrombin time (dTT) and chromogenic ecarin clotting time (ECT) have better correlation with serum concentration and would be helpful for quantitative detection of dabigatran and its clinical efficacy; however, there is limited use of this assay in most clinical scenarios, as tests await US FDA licensing [23].

- A normal thrombin time (TT) can effectively rule out presence of any significant serum level of dabigatran, though an elevated thrombin time does correlate well with the serum dabigatran level.
- For all the factor Xa inhibitors, use of anti-factor Xa activity level can reliably assess the degree of anticoagulation, both qualitatively and quantitatively, provided that the instruments are calibrated for the specific agents. It is advisable to use these calibrated anti-factor Xa activity tests, but if unavailable, then a generic chromogenic anti-factor Xa activity can rule out a meaningful level of any factor Xa inhibitor. A normal thrombin time usually helps exclude supratherapeutic anti-factor Xa inhibitor levels [24, 25].

Fortunately, since 2015, a specific reversal agent for dabigatran, idarucizumab, has been FDA-approved, with two other agents (andexanet-*α* and ciraparantag) have been fast-tracked through the FDA for potential approval for the reversal of the factor Xa inhibitors. A detailed discussion of their mechanism of action will be detailed below in the management of the intracranial hemorrhage.

## 2. Intracranial Hemorrhage Management

The emergency treatment of ICH starts with the basic care of the acutely ill patient, with an aim for stabilizing the hemodynamic condition, assessing the level of neurologic injury, and providing specific therapeutic measures and interventions if possible, along with the supportive medical management.

### 2.1. Initial Evaluation and Clinical Stabilization

Spontaneous intracranial hemorrhage, being a medical emergency, needs to be managed aggressively. Basic steps are recommended to be followed as per the guidelines from the American Heart Association and the Neurocritical Care Society [26, 27].

**Figure 2:**
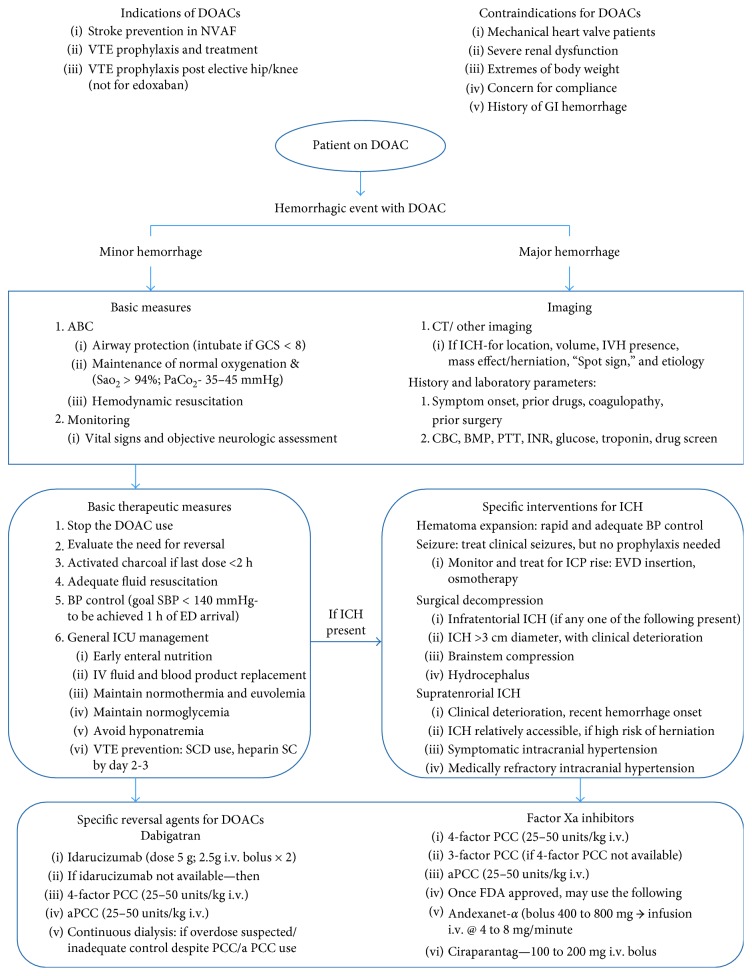
Management algorithm for DOAC-related hemorrhage [13, 14, 28, 35, 38, 44]. ABC = airway, breathing, circulation, aPCC = activated prothrombin complex concentrate, BMP = basic metabolic panel, BP = blood pressure, CBC = complete blood count, CT = computerized tomography, DOACs = direct oral anticoagulants, ED = emergency department, EVD = external ventricular drain, GCS = Glasgow Coma Scale, GI = gastrointestinal, i.v. = intravenous, ICH = intracranial hemorrhage, ICP = intracranial pressure, ICU = intensive care unit, INR = international normalized ratio, IVH = intraventricular hemorrhage, kg = kilogram, mg = milligram, NVAF = nonvalvular atrial fibrillation, PaO_2_ = partial pressure of oxygen, PCC = prothrombin complex concentrate, PTT = partial thromboplastin time, SaO_2_ = oxygen saturation, SBP = systolic blood pressure, SC = subcutaneous, SCD = sequential compression device, and VTE = venous thromboembolism.

- A summarized version of the recommendations includes immediate evaluation and stabilization of the airway, breathing, and circulation, with a focused neurologic and clinical exam for lesion location and its severity evaluation and the use of CT (computerized tomography) scan to help with this. Meticulous management of hemodynamic stability can be achieved by appropriate blood pressure medications and adequate intravenous (i.v.) fluid resuscitation, including blood product transfusion, if indicated. Concurrent optimization of blood pressure management, reversal of coagulopathy and prompt surgical intervention when indicated, is essential in decreasing morbidity and mortality. This is usually followed by intensive care management and monitoring for prevention of any untoward complications (Figure 2).

### 2.2. Poor Prognostic Factors

- Presence of coma, neck stiffness, focal neurologic deficits with seizures, diastolic blood pressure > 110 mmHg, and vomiting on presentation suggests a presence of ICH. Poor prognostic predictors in this situation are decreased level of consciousness, larger hematoma volume on presentation, and presence of intraventricular hemorrhage (IVH) [28].

Patients with ICH volume of above 60 mL (millilitres) with Glasgow Coma Scale (GCS) below 8 have a likely poor outcome (predicted 30-day mortality rate above 90%) versus patients with hematoma volume below 30 mL and GCS above 9, who have mortality rate below 20% in the same period. IVH presence is an independent poor predictor of outcome. An increase in IVH volume by more than 2 mL in the first 24 hours is associated with an odds ratio (OR) for poor outcome of 4.2 (95% CI 1.06–16.63, *p*=0.0405) [29].

### 2.3. Hematoma Location

Typical hypertensive ICH locations are basal ganglia, thalamus, deep cerebellar nuclei, internal capsule, midbrain, and pons. Lobar hemorrhages are usually associated with cerebral amyloid angiopathy, arteriovenous malformations, brain tumors, or other structural lesions. Lobar hemorrhages are typically associated with a worse outcome as compared to the usual hypertensive ICH locations [26, 27].

### 2.4. Hematoma Expansion

All the ICH patients are at risk of early hematoma expansion (HE) that may lead to further neurological deterioration. HE has been noticed in up to 40% of patients within first 24–48 hours. Most of these patients have HE within first 6 hours of the onset, with about 26% of ICH patients demonstrating substantial hematoma volume increase (>33% rise above baseline hematoma volume) within 4 hours of symptom onset. Additional 12% patients had HE within 20 hours on repeat CT scan [30, 31]. Predictors of hematoma expansion include history of warfarin/DOAC use with associated coagulopathy, shorter time from ICH onset to CT, and presence of “spot sign” on CT angiogram. Latter is due to contrast extravasation within the hematoma on postcontrast CT head. If it is detected in the arterial phase of CT angiogram, then it has higher chances of absolute HE and therefore a worse outcome [31, 32].

### 2.5. Clinical Severity Assessment

Regular use of clinical severity assessment scales helps to evaluate objectively the ICH patients, in a standardized, observer-independent manner. Of the several clinical grading scales, the ICH score is probably the most popular. It uses consciousness level (as measured by GCS or Glasgow coma scale), age, ICH volume, IVH presence, and ICH location (supratentorial/infratentorial) to generate a score between 0 and 6; the higher the score, the more the mortality rate. However, these clinical grading scales should never be used in isolation, for deciding the acute initial management of ICH patients.

### 2.6. Medical Management of ICH

Acute management and monitoring of ICH patients should preferably be done in dedicated neuroscience critical care units or dedicated stroke units, to positively impact the outcome and mortality rates. Essential monitoring of neurologic and hemodynamic parameters with the use of intracranial pressure-monitoring devices, multimodal neuromonitoring should be provided, as needed.

Several clinical trials (INTERACT 2 and ATACH 2) have established the safety of early intensive blood pressure reduction. Rapid intensive blood pressure lowering has been shown to decrease the chances of hematoma expansion, particularly with larger hematomas/positive spot sign on contrast-CT, though with systolic BP level below 130 mmHg, there may be complications, especially related to renal function [32, 33]. It is equally important to maintain tight control over several important clinical and physiological parameters, such as prevention of venous thromboembolism (VTE), infections, and seizure control and prophylaxis. Maintenance of normothermia (goal core body temperature < 37.5°C) and normoglycemia (serum glucose between 140 and 180 mg/decilitre) is also recommended for critically ill patients.

Close monitoring for possible intracranial hypertension, and aggressive management of the same, if detected, is critical. Its management involves simple measures such as head-of-bed elevation, neck positioning in midline, avoidance of unnecessary noxious stimuli, with adequate analgesia and sedation, and maintenance of normal temperature and ventilation to complex interventions such as hyperosmolar therapy, ventriculostomy placement, medically induced coma with use of barbiturates or therapeutic hypothermia, and decompressive craniectomy (Figure 2) [26, 27].

### 2.7. Reversal of Anticoagulant Medications

The use of antiplatelet medications is also associated with increased incidence of the hemorrhagic complications, including ICH. Use of platelet transfusion to reverse the antiplatelet activity is controversial, with lot of variation in the daily practice between the institutions. Based on the limited evidence about the use of platelet transfusion to reverse the effect of irreversible antiplatelet medications (such as aspirin), there is no benefit in platelet transfusion in patients with aspirin resistance (as can be found by platelet function assays), in those with normal platelet function, and in those who are not undergoing neurosurgical procedure. If there is a need for neurosurgical procedure and when platelet function assay is not available, then transfusion may be reasonable [26].

For patients on warfarin with INR above 1.5, the current guidelines recommend the use of 4-factor PCC (prothrombin complex concentrate) that effectively reverses the warfarin within 30 minutes of its use [34]. The PCC is a donated blood byproduct that contains virally inactivated concentrated plasma coagulation factors.

For patients on DOAC use, there was no specific reversal agent available until the approval of idarucizumab in October 2015, for dabigatran reversal. Subsequently, there has been fast-tracking for FDA approval of another 2 candidate medications for reversal of factor Xa inhibitors, andexanet-*α* and ciraparantag, and the latter can also reverse the anticoagulant effects of unfractionated heparin and low-molecular weight heparin, in addition to that of the factor Xa inhibitors (Table 2).

### 2.8. Idarucizumab

Idarucizumab (Praxbind) is a fully humanized Fab fragment of monoclonal antibody against dabigatran, with 350-fold higher binding affinity for dabigatran than for thrombin. It is cleared renally, and it rapidly and completely reverses the anticoagulant action of dabigatran, with no prothrombotic activity of its own. It can be used multiple times if needed, without loss of activity. It has not shown any evidence of significant immunogenicity. It does not affect other anticoagulants and does not activate clotting despite its structural resemblance to thrombin. It has been approved for dabigatran reversal in emergent surgery, urgent procedures, or life-threatening and/or uncontrolled bleeding including ICH.

The recommended dose is 5 g (given as 2 consecutive infusions of 2.5 g vials within 15 minutes interval). The anticoagulant effect of dabigatran may reappear after 12–24 hours after idarucizumab use due to dabigatran redistribution from the tissues into the plasma, and idaruiczumab may have to be repeated to maintain the normal coagulation profile. Similarly, it may be administered again if excessively high dabigatran concentration is present, as in cases of overdose [37, 45].

Idarucizumab is not affected by renal or hepatic dysfunction and is reported to have delirium, headache, and constipation as common side effects. Onset of activity is within minutes of idarucizumab administration, and hemostasis is restored in a median of 11.4 hours, with duration of effect lasting at least 24 hours. Its metabolites are excreted in urine within the first few hours. Dabigatran can be restarted within 24 hours after idarucizumab use, if indicated [44]. The REVERSE-AD study (reversal effects of idarucizumab on active dabigatran; Clinicaltrials.gov NCT02104947) studied 504 patients on dabigatran needing urgent reversal due to major bleeding event or due to the need for emergent surgery or procedure. This was reversed with the use of idarucizumab (5 g). After its administration, at 4 hours, the median maximum reversal was 100% for the diluted thrombin time (dTT), ECT (ecarin clotting time), and aPTT. In the procedural group, 93% of patients had normal periprocedural hemostasis. It also normalized conagulation tests to the same extent in ICH cases, as it did in other major bleeding event cases [14, 37, 39, 46].

Few case reports have demonstrated that idarucizumab may be used to reverse the dabigatran if the patient has an acute ischemic stroke while on dabigatran. This small review reported 21 patients who had mild to moderate ischemic stroke while on dabigatran, with the use of idarucizumab to reverse the formers' effect. This was followed by administration of tissue plasminogen activator (tPA) in 18 patients. An unfavorable outcome was present in 3/19 patients (16%), with one fatality from symptomatic postthrombolysis intracranial hemorrhage and worsening of ischemic stroke in other 2 patients. Systemic bleeding, venous thrombosis, or allergic reactions was not noticed. The suggested thresholds for i.v. thrombolytic therapy that can be performed safely in dabigatran-treated patients are TT (below 38 seconds) or aPTT (below 37 seconds) [42, 47, 48].

A concern has been raised regarding significant delay in cessation of bleeding by idarucizumab in dabigatran-associated intracranial hemorrhage. It is unclear if the blood-brain barrier has a role in ease of access to the bleeding site by idarucizumab. Adding blood component therapy (e.g., PCC and/or activated PCC) along with idarucizumab may be helpful till we have more robust clinical data [49].

### 2.9. Andexanet Alpha

Andexanet-*α* (PRT064445) is a catalytically inactive recombinant form of factor Xa that is derived from Chinese hamster ovarian cells. This Xa mimetic molecule serves as a “decoy” for the Xa anticoagulants by acting as a competitive inhibitor for the native factor Xa. In essence, andexanet-*α* diverts anticoagulants away from its intended target, the factor Xa. Though andexanet-*α* was designed to work against rivaroxaban, apixaban and edoxaban by binding to the above drugs in 1 : 1 ratio, it also binds various forms of heparin, including unfractionated heparin, low-molecular weight heparin, as well as, fondaparinux. The latter action is by competitive binding to the antithrombin-heparin complex. It therefore reverses the indirect factor Xa inhibitors, direct factor Xa inhibitors, and also the heparin and low-molecular weight heparin via its effect on the anti-factor Xa and anti-factor IIa (thrombin) activity of the heparins due to its noncovalent interaction with the antithrombin-heparin complex.

Being similar to factor Xa, it also binds to the tissue factor pathway inhibitor (TFPI), reducing TFPI activity, but unlike native factor Xa-TFPI complex, the andexanet-TFPI complex fails to inhibit the factor VIIa-tissue factor complex. Consequently, andexanet-α administration in a patient on factor Xa inhibitors may develop a transient procoagulant state by this mechanism. The clinical significance of this interaction, however, remains to be clarified.

Andexanet has an initial half-life of approximately 15 minutes, with terminal half-life of approximately 6 hours after intravenous infusion. It is supplied in vials of 100 mg of lyophilized drug that remain stable for 2 years with refrigeration. The antidote needs to be reconstituted with sterile water for intravenous infusion. Reconstituted drug is stable for at least 8 hours at room temperature. A low-dose regimen typically needs 9 vials, and a high-dose regimen needs a total of 18 vials. Typically, it is administered as an initial bolus (400 or 800 mg), and then the remainder of the drug is infused over next 2 hours (at 4 mg to 8 mg/minute) [12].

In ANNEXA-4 study (ability of andexanet-*α* to reverse the anticoagulant activity study; Clinicaltrials.gov-NCT02329327), 47 patients were medicated with either rivaroxaban or apixaban for the treatment of atrial fibrillation or VTE; subsequently, they were treated with andexanet-*α* for high anti-factor Xa activity. 66% patients were found to have excellent hemostasis, with anti-factor Xa activity reduction for several hours following its use. 18% patients developed thromboses (5 strokes and 8 VTEs) within 30 days after andexanet treatment. All of them, except one, were not receiving therapeutic anticoagulation at the time of the adverse event [40]. Andexanet-*α* is not yet FDA-approved for use.

### 2.10. Ciraparantag

Ciraparantag (PER977) is a synthetic small molecule antidote that has a charge-dependent binding to the heparins, as well as, a hydrogen bond-mediated interaction with DOACs, thus, preventing both old and new anticoagulant classes of medications from binding to their endogenous targets. It potentially can work as a universal anticoagulant antidote, providing activity against direct thrombin inhibitors, factor Xa inhibitors, heparins (including low-molecular weight heparin), and fondaparinux. Metabolites of ciraparantag are rapidly eliminated through the kidneys. Unfortunately, routine coagulation tests cannot monitor the reversal effect of ciraparantag. Although the “whole blood-clotting time” can be used to monitor its effect, this test has limited availability.

Ciraparantag was able to reverse the effect of therapeutic enoxaparin (at doses of 200 mg and 100 mg within 5 and 20 minutes, respectively, following the enoxaparin dose. This was a phase I/II study of 40 healthy volunteers [41]. In another phase I/II study of 80 healthy volunteers receiving edoxaban, ciraparantag demonstrated dose-dependent reversal of whole blood-clotting time to within 10% of the baseline [43].

Though a single bolus is sufficient to reverse the effects of enoxaparin or edoxaban, ciraparantag can be repeated if needed. The side effects include transient perioral and facial flushing, abnormalities in taste sensation, and headache. This will be supplied in vials of 300 mg that is stable for 2 years at room temperature [12]. Ciraparantag is not yet FDA-approved.

Another novel anticoagulant antidote under development is FXa^I16L^, a mutant factor Xa that has leucine substituting for isoleucine at position 16. It has a potential use as a universal bypassing agent for multiple anticoagulants. It circulates in a zymogen-like (inactive) state in plasma, without binding to any anticoagulant and is resistant to active-site inhibitors. It is activated when it comes across activated factor V (factor Va) on damaged cellular surfaces, leading to selective restoration of hemostasis at the bleeding site. FXa^I16L^ has demonstrated reversal of rivaroxaban in a mouse model and the reversal of rivaroxaban and dabigatran in human plasma in vitro [50]. Results of further studies on this potential drug are eagerly awaited.

### 2.11. Nonspecific Reversal Agents

DOACs can be reversed at least in part by the use of activated charcoal, provided that the last dose was ingested within 2 hours. Hemodialysis can be used for dabigatran-related hemorrhagic states, particularly in cases of overdose, due to its renal-dependent clearance.

## 3. Prothrombin Complex Concentrate (PCC)

3- and 4-factor PCC are now commonly available and used for the reversal of vitamin K antagonists. For warfarin-related hemorrhage, the 4-factor PCC is the recommended reversal agent. 3-factor PCC contains the vitamin K-dependent coagulation factors, namely, factor II, IX, and X, with a minimal amount of factor VII, whereas the 4-factor PCC has a proportionally larger amount of factor VII compared to factor IX.

The 4-factor PCC has demonstrated marginal utility in the reversal of all of the recent factor Xa inhibitors (rivaroxaban, apixaban, and edoxaban). The usual dose found to be effective is 50 units/kilogram for restoring normal bleeding time and reestablishing thrombin generation. Currently, the guidelines recommend PCC as the treatment of choice for patients taking DOACs presenting with ICH. This applies for both factor Xa inhibitors and for dabigatran if idarucizumab is not available [51].

### 3.1. Activated PCC

Activated PCC (FEIBA or factor eight inhibitor bypassing activity) contains the regular 4-factor PCC, but unlike the PCC, factor VII is in the activated state, with the usual dose being 50 units/kilogram. This medication has shown to effectively control the ICH expansion in a small prospective trial of 127 ICH cases, with 6 patients on DOACs [52].

The current guidelines recommend the use of FEIBA (activated PCC) for reversal of factor Xa inhibitors (rivaroxaban, apixaban, and edoxaban) only when the PCC fails, as activated PCC was not found to be superior to PCC for this indication, and carries a higher risk of thrombotic complications than regular PCC [27].

### 3.2. Recombinant Factor VIIa (rFVIIa)

Though the utility of rFVIIa in control of hematoma expansion in a mouse model has been proven, but due to lack of strong human data and elevated thrombotic risk, the use of rFVIIa is not recommended currently with DOACs related ICH, unless other measures have failed. The usual dose used is 90 micrograms/kilogram body weight.

### 3.3. Practical Considerations during Reversal of DOACs

DOAC reversal may not be necessary if the last dose was taken at least 48 hours prior to the hemorrhagic event, but likely if associated severe renal or hepatic dysfunction exists. With limited information regarding dosing, rapid quantitative serum levels of DOAC may be helpful. DOAC levels exceeding 30 nanograms/mL require reversal of the DOACs. Direct thrombin inhibitor (dabigatran) can be reversed by the antibody, Idarucizumab, (two boluses of 2.5 grams each within 15 minutes) or by administering a 4-factor PCC (50 units/kilogram dose) if this specific antidote is unavailable.

### 3.4. Reinitiation of the DOACs after ICH

Though the annual risk of any major bleed from oral anticoagulant (OAC) use is 2-3%, with OAC-related ICH risk of 0.3–0.5%, the annual risk for thromboembolic complications is much higher in the absence of OAC therapy, in patients where it is indicated. The annual arterial thromboembolic complication risk for patients with mechanical heart valves is 12% to 22%, atrial fibrillation with CHA_2_DS_2_-VASc score of above 3 is 6% to 18%. To help with the clinical decision-making, there have been several scoring systems devised, but they may provide an inadequate ability to differentiate between a major bleeding event and clinically relevant nonmajor hemorrhage predictability [53]. Two major factors that guide the clinical decision-making about the reinitiation of the DOACs after ICH are indications for DOAC use and the predicted risk of VTE/ stroke versus the risk of hemorrhage associated with it. Factors that favor restarting OAC therapy are location of ICH (deep ICH), presence of mechanical heart valve, secondary prevention of acute ischemic stroke, high risk of stroke or VTE, and a corrected cause of potential bleeding (e.g., a clipped aneurysm or repaired vascular malformation). Factors that demonstrate higher risk of hemorrhagic complications include lobar ICH and imaging suggestive of cerebral amyloid angiopathy (multiple microbleeds on gradient-echo magnetic resonance imaging) [28].

The next important decision would be the timing of reinitiation of the OAC therapy. In patients with mechanical heart valve or stable gastrointestinal bleed, the OAC therapy is restarted earlier, as compared to patients with ICH or low risk of stroke/VTE. On the other hand, in patients with lobar ICH, it may not be safe to restart OAC therapy at all. For deep ICH and high risk of cerebral ischemia (e.g., mechanical heart valve/ atrial fibrillation with high CHA_2_-DS_2_-VASc Score), the OAC treatment should be restarted within 1-2 weeks and even later (after 4 weeks) if the risk of hemorrhage is higher. It is important to note that DOACs reach therapeutic anticoagulation level within a few hours, unlike VKAs that need a few days to do so.

## 4. Conclusion

Spontaneous ICH remains an important cause of mortality amongst the patients with stroke. Its mortality rate has unfortunately not improved in the last several years. Oral anticoagulant medications account for a small but increasingly common cause of this dreaded complication. DOACs are likely to further contribute to this neurological emergency due to their rising popularity. Most of the hemorrhagic complications from DOACs can be managed without any specific reversal agents. Presence of the rare ICH in a patient on DOAC, however, warrants immediate reversal of anticoagulation, and, therefore, all the hospitals should have a management protocol in place for hemorrhagic complications due to DOACs. Comprehensive information about DOACs, their specific reversal agents, and recommended dosing, along with information on supportive measures, need to be made available to all the staff who are involved in the management of such patients, especially those with ICH.

DOAC use is likely to become more prevalent with the improved understanding of growing indications and increased availability and development of the specific reversal agents. However, this needs to be supplemented by prepared emergency response system for these rare hemorrhage-related events from DOAC complications with a management protocol based on a multidisciplinary team approach. A collaborative, efficient, and effective strategy, that has the flexibility of adapting to the ongoing developments in diagnostic tests for DOACs, as well as, invention of better and broader spectrum antidote agents, must be developed and implemented for the best patient care.

## Figures and Tables

**Table 1:** Properties of different direct oral anticoagulants (DOACs) [8, 13–15].

	Dabigatran	Rivaroxaban	Apixaban	Edoxaban	Betrixaban
Mechanism of action	Direct thrombin inhibitor	Factor Xa inhibitor	Factor Xa inhibitor	Factor Xa inhibitor	Factor Xa inhibitor
Time to peak serum level	1 hour, 2 hours with food	2–4 hours	3-4 hours	1-2 hours	3-4 hours
Elimination half-life (hours)	12–17 (young), 14–17 h (in elderly)	5–9 (young), 11–13 (in elderly)	12 (8–15)	10–14	19–27
Elimination	80% renal	70% liver, 30% renal	30% renal	50% renal	11% renal, 89% fecal
Bioavailability	3%–7%	66%–100% higher with food	50%	62%	34%
Dose/frequency NVAF	150 mg bid (110 mg bid, if age > 80 years)	20 mg once daily	5 mg bid	60 mg once daily	Not licensed
VTE therapy and prophylaxis	150 mg bid (after 5 d of LMWH)	15 mg bid × 21 d, then 20 mg once daily	10 mg bid × 7 d, then 5 mg bid	60 mg bid (after 5 d of LMWH)	160 mg on day 1, then 80 mg daily
VTE prophylaxis post elective hip/knee surgery	150 mg once daily	10 mg once daily	2.5 mg bid	Not licensed	Not licensed
P-gp resecretion	Yes	Yes	Yes	Yes	Yes
CYP3A4 metabolism	No	Yes	Yes	Minimal	No
Follow-up monitoring	Renal function, CBC periodically, at least annually	Renal function, CBC periodically, at least annually; hepatic function	Renal function, CBC periodically, at least annually	Renal function, CBC periodically, at least annually	Renal function, CBC periodically, at least annually
Quantitative assay	Dilute thrombin time (dTT), ecarin clotting time (ECT)	Specific, calibrated anti-FXa assays	Specific, calibrated anti-FXa assays	Specific, calibrated anti-FXa assays	Specific, calibrated anti-FXa assays

Anti-FXa assay = anti-factor Xa assay, bid = twice daily, CBC = complete blood count, CYP3A4 = cytochrome P450 3A4, d = day, DOACs = direct oral anticoagulants, FXa = factor Xa, INR = international normalized ratio, LMWH = low-molecular weight heparin, mg = milligram, NVAF = nonvalvular atrial fibrillation, P-gp = P-glycoprotein, PTT = partial thromboplastin time, and VTE = venous thromboembolism.

**Table 2:** Properties of specific reversal agents for use against the DOACs [21, 35–37, 39–41].

	Idarucizumab	Andexanet-α	Ciraparantag
Target	Dabigatran	Factor Xa inhibitors, LMWH, fondaparinux	Factor Xa inhibitors, LMWH, fondaparinux, heparin, and dabigatran
Compound	Humanized monoclonal antibody fragment	Modified recombinant derivative of human FXa (inactive)	Synthetic small molecule
Mechanism of action	350x higher affinity binding to dabigatran than dabigatran-thrombin-binding affinity	“Decoy” receptor for FXa inhibitor with higher binding affinity than natural FXa	Binds to target via noncovalent hydrogen bonds and charge-charge interactions preventing anticoagulants from binding to endogenous targets
Dose	5 g (as sequential i.v. boluses of 2.5 g each)	210–420 mg i.v. bolus + 2 h i.v. infusion at 4–8 mg/min	100–400 mg i.v. bolus
Onset of action	Immediate	Within 5 minutes	Within 10 minutes
Duration of reversal	12 hours	1-2 hours	24 hours
Elimination	Renal	Unknown	Unknown
Clinical trial	REVERSE-AD [35, 36]	ANNEXA-A [42] ANNEXA-R [42]	Ansell et al. [43]
Developmental phase	III/approved	III	II
Storage/stability	Refrigerated/2 years	Refrigerated/2 years	Room temperature/2 years
Side effects	Injection site skin reaction and hematoma, epistaxis	Urticarial, flushing, dysgeusia, headache	Flushing, dysgeusia, headache

FXa = factor Xa, g = grams, i.v. = intravenous, and mg = milligram.
